# Vitamin D: Not Just Bone Metabolism but a Key Player in Cardiovascular Diseases

**DOI:** 10.3390/life11050452

**Published:** 2021-05-18

**Authors:** Marcello Izzo, Albino Carrizzo, Carmine Izzo, Enrico Cappello, Domenico Cecere, Michele Ciccarelli, Patrizia Iannece, Antonio Damato, Carmine Vecchione, Francesco Pompeo

**Affiliations:** 1 Department of Mathematics for Technology, Medicine and Biosciences Research Center, University of Ferrara, 44121 Ferrara, Italy; 2 Specialist Medical Center-Via Cimitile, 80035 Nola, Italy; 3 IRCCS Neuromed, 86077 Pozzilli, Italy; albino.carrizzo@gmail.com (A.C.); enrico.cappello@gmail.com (E.C.); domenicocecere71@gmail.com (D.C.); antonio.damato85@gmail.com (A.D.); cvecchione@unisa.it (C.V.); pompeo@neuromed.it (F.P.); 4 Department of Medicine, Surgery and Dentistry, University of Salerno, 84081 Baronissi, Italy; carmine.izzo93@gmail.com (C.I.); mciccarelli@unisa.it (M.C.); piannece@unisa.it (P.I.)

**Keywords:** vitamin D, cardiovascular diseases, metabolic diseases

## Abstract

Vitamin D is the first item of drug expenditure for the treatment of osteoporosis. Its deficiency is a condition that affects not only older individuals but also young people. Recently, the scientific community has focused its attention on the possible role of vitamin D in the development of several chronic diseases such as cardiovascular and metabolic diseases. This review aims to highlight the possible role of vitamin D in cardiovascular and metabolic diseases. In particular, here we examine (1) the role of vitamin D in diabetes mellitus, metabolic syndrome, and obesity, and its influence on insulin secretion; (2) its role in atherosclerosis, in which chronic vitamin D deficiency, lower than 20 ng/mL (50 nmol/L), has emerged among the new risk factors; (3) the role of vitamin D in essential hypertension, in which low plasma levels of vitamin D have been associated with both an increase in the prevalence of hypertension and diastolic hypertension; (4) the role of vitamin D in peripheral arteriopathies and aneurysmal pathology, reporting that patients with peripheral artery diseases had lower vitamin D values than non-suffering PAD controls; (5) the genetic and epigenetic role of vitamin D, highlighting its transcriptional regulation capacity; and (6) the role of vitamin D in cardiac remodeling and disease. Despite the many observational studies and meta-analyses supporting the critical role of vitamin D in cardiovascular physiopathology, clinical trials designed to evaluate the specific role of vitamin D in cardiovascular disease are scarce. The characterization of the importance of vitamin D as a marker of pathology should represent a future research challenge.

## 1. Introduction

The history of vitamin D started in 1919 when it was pointed out, by Huldschinsky, that children with rickets healed when exposed to ultraviolet light [1]. Since then, many things have changed, but while its role in the absorption of calcium and bone metabolism is now known, the other actions of vitamin D are still yet to be thoroughly investigated. The possible wide range of new therapeutic roles of vitamin D has opened up novel therapeutic perspectives. In fact, in addition to its role as a fat-soluble vitamin, vitamin D is also a prohormone. This unique feature is accompanied by the fact that vitamin D stores do not rely exclusively on dietary intake [2].

The vitamin D receptors (nuclear receptor—VDRs) are present in several tissues other than the intestine and bones, such as the brain, breast, prostate, lymphocytes, etc. Indeed, recent research showed that higher levels of vitamin D might protect against various pathologies: diabetes mellitus, osteoporosis, osteoarthritis, hypertension, cardiovascular diseases, metabolic syndrome, depression, various autoimmune diseases, neoplasms of the breast, prostate, and colon, etc. [3,4]. Therefore, it is essential to examine the actual bone and extra-bone therapeutic potential of vitamin D [5]. Current knowledge seems to show that the dosage of vitamin D should enter more into the clinical practice of preventive medicine to implement a correct oral integration in the case of deficiencies to improve the state of health [6]. With doses up to 100,000 IU/month for adults, vitamin D supplementation is clinically safe and physiologically reasonable, since these doses are consistent with physiological needs [7]. Monthly supplementation increases the serum concentration of vitamin D, but no significant effects on fractures, bone mineral density, and CVD have been highlighted so far [4,8]. Periodic evaluation of serum 25-OH-vitamin D (25 (OH) D) or 25-hydroxy-vitamin D, in addition to calcium and serum parathormone (PTH), can help optimize the serum levels of vitamin D for better disease prevention and treatment.

Despite controversies and uncertainties, the prevalence of vitamin D deficiency in patients is high, and treatment evaluations are needed [8]. In this review, we examined the role of vitamin D in different pathophysiological contexts and other disease settings.

### Pathophysiological Bases of Vitamin D

Vitamin D is naturally obtained mainly from two possible sources, either sunlight or dietary intake. Vitamin D3 (cholecalciferol of animal origin) is the type of vitamin D produced in the skin (starting from 7-dehydrocholesterol after sun exposition) and nutrition (fatty fish, cod liver oil, etc.). Vitamin D2 (ergocalciferol) has a vegetable origin and is obtained from the ultraviolet irradiation of ergosterol, present in many mushrooms. The content of vitamin D2 in mushrooms and the lichen Cladina arbuscula increases with exposure to ultraviolet light. This process can be emulated by industrial ultraviolet lamps, increasing the levels of vitamin D2 produced by irradiated mushrooms.

Nevertheless, vitamin D2 is less efficient as a biological precursor to the final active form of the biologically active vitamin D, “1,25-dihydroxyvitamin or calcitriol”. Ergocalciferol (vitamin D2) has a worse pharmacokinetic profile than D3, and it can be contaminated during its microbial production. With this, vitamin D2 is potentially less effective and more toxic than cholecalciferol (vitamin D3) [9]. Consequently, cholecalciferol is by far the preferred form of supplementation in the world. The prohormone calcidiol, produced in the liver by hydroxylation of cholecalciferol (vitamin D3), is converted in the kidneys by the enzyme 25-hydroxy vitamin D3 1-alpha-hydroxylase in calcitriol, which is a biologically active secosteroid hormone of vitamin D3 (cholecalciferol).

Other authors recently correlated the effects of some statins and vitamin D levels in patients with coronary artery disease [10]. A recent meta-analysis showed that the integration of vitamin D, 1000 IU/day, can correct the high serum PTH levels present in overweight/obese patients, while, in the same patients, 4000 IU/day of vitamin D caused a significant increase in the serum levels of vitamin D (of 25-OHD) [11]. Similarly, the relationship between vitamin D/PTH in cardiovascular pathologies has been studied in numerous other reports in recent years. Recent works have shown the synchronicity of action between vitamin D and omega-3 fatty acids (EPA-eicosapentaenoic acid and DHA-doicosahexaenoic acid, which can be formed from the precursor AC-linolenic acid (ALA), known to be found in foods such as legumes, walnuts, soybean oil, salmon, tuna, bluefish, etc.) [12]. Vitamin D in its 25-hydroxycholecalciferol form (calcidiol or calcifediol), generated by hepatic transformation (enzyme 25-hydroxylase) of what is produced by the skin by solar radiation (cholecalciferol starting from its precursor 7-dehydrocholesterol), can also be absorbed by numerous cell types. In practice, all cells equipped with 1α-hydroxylase (an enzyme that synthesizes the biologically active vitamin D in the kidney, calcitriol) and equipped with the nuclear receptor of vitamin D (the receptor of vitamin D or calcitriol or NR1I1 of the VDRs gene is a nuclear receptor capable of activating various transcription factors). Therefore, these cells can independently produce their calcitriol without having to rely on replenishment through renal activation. In this way, many cells and tissues containing the enzyme 1α-hydroxylase can independently produce the biologically active form of vitamin D, “calcitriol”. This aspect has contributed to changing the way in which we understand the activities of vitamin D. Numerous cells are capable of extra-renal calcitriol self-production (breast, lung, prostate, skin, colon, pancreas, lymph nodes, adrenal gland, endothelium, cerebellum, and cerebral cortex, lymphocytes, macrophages, skin, etc.) (Table 1).

Calcitriol can also modulate the transcription of numerous genes (genomic response) and, in particular, those that operate in cell differentiation–proliferation such as c-myc, c-fos, and c-sis in the delicate balance between oncogenes (genes facilitating multiplication and the growth of neoplastic cells) and antioncogenes or tumor suppressor genes [13]. Other reports concern the modulation actions of neurotransmitters (antidepressant-anticonvulsant action) [14], regulatory actions for immunity [15], inflammation [16], microbiota [17], infections [18], making calcitriol a pleiotropic substance [19]. Calcitriol, as a secosteroid hormone, recalls the other steroid hormones (estradiol, progesterone, testosterone, cortisol, and aldosterone, etc.) with two fundamental actions: a slow one (slow genomic response) and a faster one (rapid non-genomic response), such as the control of calcium-phosphorus absorption. Both responses are mediated by VDRs’ nuclear receptors, similarly to steroid hormones. However, these VDRs are also present outside the nucleus (cytoplasm or inner face of the plasma) [20].

Through interaction with VDR cell receptors, circulating levels of calcitriol self-regulate its renal biosynthesis (high levels with negative feedback and vice versa), while low circulating parathormone (PTH) levels stimulate renal biosynthesis of calcitriol.

The VDR is part of the vast family of nuclear receptors (NR:), a superfamily of transcription factors [21]. The large family of nuclear receptors work as “sensor-ligands” capable of binding fat-soluble hormones such as steroids, retinoids, thyroid hormones, vitamin D, fatty acids, oxysterols, and numerous xenobiotics (from the Greek consisting of ξένος -η -ον “xènos-and -on” = foreigner and βίος “bìos” = life). Whether natural or synthetic, these substances are almost always lipophilic, exogenous, and can function both as a drug or a toxin (such as antibiotics, etc.). The nuclear receptor for steroids is also called a “xenobiotic sensor”, as it can interact with numerous xenobiotics. This receptor can crosstalk with the VDR by altering vitamin D metabolism, especially in the kidney, liver, and intestine [22]. The crosstalk between SXR and VDR may be responsible for the toxicity of various xenobiotics. The role of vitamin D in bone metabolism has long been known, and its deficiency can lead to reduced absorption of intestinal calcium. When the blood level of vitamin D is sufficient, the absorption rate of calcium in the intestine is 30–40%. Vice versa, if the vitamin level is low, the absorption drops to less than 10–15% [23]. Many RCTs have shown that vitamin D-calcium supplementation reduces the risk of bone fractures [24], but its role in health, tropism and muscle efficiency is also reported with postural improvement and reduced risk of falls, in contrasting elderly sarcopenia, visceral fat and improving the HOMA index [25].

Another critical aspect is discovering the vitamin D carrier protein [26], called vitamin D-binding protein (DBP). DBP is a protein (glycosylated alpha-globulin weighing ~ 58 kDa) which in humans is synthesized by liver parenchymal cells and secreted in the bloodstream. It is encoded by the CG (protein coding) gene. DBP is capable of shuttling the metabolites of vitamin D between the skin, liver, and kidneys and in all organs and tissues, and also intervenes in response to pathogens such as viruses and bacteria. Overall, 99% of the different lipophilic metabolites of vitamin D are transported in circulation by plasma proteins, the most important of which is vitamin D-binding protein (DBP), capable of binding with a high decreasing affinity 25 (OH) D/24.25 (OH) 2D/1,25 (OH) 2D. The 25 (OH) D linked to DBP (calcidiol or 25-hydroxycalciferol) is filtered by the kidneys, captured by the cells of the proximal tubules through endocytosis, and hydroxylated by ‘1-alpha-hydroxylase in the biologically active form of vitamin D (calcitriol). DBP is then subsequently degraded. The circulating complexes of the lipophilic metabolites of vitamin D linked to the carrier protein (DBP) have limited access to target cells and tissues, and therefore the biological activity of vitamin D is linked to the free portion of the vitamin. DBP has the fundamental task of buffering any high levels of free and biologically active vitamin D, thus avoiding any intoxication [27]. DBP polymorphisms are very frequent, so much so that more than 120 have been described (gene coding for GC/DBP). Still unknown are the possible consequences of DBP polymorphisms on the state of health/disease. Much is to be elucidated and standardized in the matter of dosage to better understand the pathophysiology of vitamin D. The half-life of DBP in human plasma is approximately 1.7 days. This is significantly shorter than the half-life of 25OHD (estimated in about 15 days with deuterium methods) [27]. The estimated daily production of DBP is around 700–900 mg/day for an adult person (10 mg/kg/day). In comparison, the body’s total albumin is approximately 280 g for a typical adult and 300 times greater than DBP. About 40% of albumin is intravascular, while the remaining 60% is interstitial (mainly muscles, adipose tissue, connective tissue, skin, etc.), with an average interstitial value of about 60–70% of the plasma value [27].

Recent findings still have not established whether calcifediol or calcitriol is the form of vitamin D of choice in clinical practice. Cholecalciferol has a more extended scientific positive history and is the most used form today in clinical practice [27]. Thanks to its pharmacokinetic properties, cholecalciferol guarantees exact dosing and prolonged administration, facilitating treatment adherence [27]. Other studies have highlighted that calcifediol is more potent and has a better intestinal absorption capacity, resulting in lower dosing and being efficacious in patients with liver failure or intestinal malabsorption [27].

## 2. Role of Vitamin D in Diabetes Mellitus, Metabolic Syndrome and Obesity

As mentioned, about 36 different types of cells are equipped with the enzyme 1-α-hydroxylase and are therefore capable of forming independently (without the need for renal activation) the hydroxylated form 1–25 of vitamin D (calcitriol) considered a real hormone. Pancreatic β cells (insulin-producing) are equipped with both the 1-α-hydroxylase and the VDR. This has opened up new pathophysiological frontiers, in particular between diabetes and vitamin D, as well as in other diseases’ pathophysiology, although so far, no substantial causal effect has been established. There is considerable evidence that suggests a role for vitamin D in the insulin secretion mechanism mediated by the vitamin D–calcium interaction. It has been shown both in vitro and in vivo that vitamin D is essential for regular insulin release in response to increased blood sugar and thus glucose tolerance [27]. Vitamin D deficiency causes a reduction in insulin secretion without altering glucagon secretion [27]. In subjects with vitamin D deficiency, vitamin D supplementation leads to improved insulin secretion and glucose tolerance (89). In humans, vitamin D supplementation improves insulin secretion in response to oral glucose in patients with mild or early type II diabetes, prediabetes, and healthy subjects, but not in well-established full-blown diabetes [28,29]. Studies do not support vitamin D supplementation as a prevention treatment for DM [30,31].

Metabolic syndrome (X syndrome, insulin resistance syndrome, or Reaven’s syndrome) is a series of interdependent risk factors that determine an increase in cardiovascular disease and mortality. In this condition, several metabolic anomalies are distinguished, such as central (visceral) obesity, dyslipidemia, insulin resistance, high blood pressure, and endothelial dysfunction. The metabolic syndrome consists of cardiovascular risk factors that synergize, increasing the atherogenic risk [32]. The expression of VDRs has also been identified by employing mRNAs in visceral adipose tissue and subcutaneous adipose tissue in both lean and obese subjects, which implies a likely regulatory role in the physiology of adipose tissue. Recent studies [33,34] highlight the role of vitamin D in adipogenesis (formation of mature adipocytes), in lipogenesis (synthesis and storage of fatty acids), and lipolysis (hydrolysis of triglycerides), as well as having an anti-inflammatory role, demonstrated in vitro and in animals, with the modulation of proinflammatory cytokines and adipokines [35,36,37]. Obesity is often associated with a reduction in circulating levels of vitamin D (it is seized by fat due to its lipophilia). This chronic deficiency could be involved in the development of insulin resistance. Several studies have shown both a positive correlation with the Quantitative Insulin Sensitivity Check Index (QUICKI), used as an insulin sensitivity indicator, and a negative correlation with the Homeostasis Model Assessment (HOMA) index, used for the degree of insulin resistance [38,39]. The controversy regarding the vitamin D–metabolic syndrome–insulin resistance relationship is an open and much-debated issue. Some studies do not seem to support this hypothesis, unlike others [40,41]. Recently, in a double-blind, randomized clinical trial (RCT) [41], albeit on a modest sample (44 patients enrolled for 3 months), insulin resistance, glucose homeostasis, and metalloproteases (MMP-9) were evaluated in obese subjects (BMI: 30–40 kg/m^2^) with a deficiency of vitamin D (25 (OH)-D: ≤50 nmol/L; ≤20 ng/mL) in which, with double-blind randomization, a high dose of vitamin D3 (50,000 U/week) was administered for 3 months together with a weight loss diet. The primary endpoints were morning fasting glucose, insulin resistance and insulin sensitivity (HOMA-IR and QUICKI), and metalloproteinases (MMP-9). The secondary endpoints were weight changes (BMI), and calcium-phosphorus metabolism, circulating vitamin D levels, calcium/phosphorus, and parathyroid hormone (PTH), also observing the possible exposure to the sun and the intake of particular foods. The final data of the study seem to indicate that the improvement in circulating levels of vitamin D (patients treated with vitamin D supplementation) resulted in a significantly greater reduction in both body weight and MMP-9 compared to the placebo group, despite the low-calorie diet being the same in the two groups. According to the authors [41], these findings are sufficient to justify wider RCT and further studies. Conflicting evidence is the main problem, as meta-analyses yield different results, failing to show a significant effect from vitamin D supplementation [42,43].

Finally, the role of vitamin D has also been reported in a possible predisposition to developing autoimmune diseases such as type I diabetes, where three key genes seem to be involved (DHCR7, CYP2R1, CYP27B1) [44]. These three genes regulate vitamin D (25 (OH) D) metabolism and are likely involved in the risk of developing type 1 diabetes. All this could establish a possible genetic etiological role of vitamin D in a predisposition to type 1 diabetes as well as in other autoimmune disorders such as multiple sclerosis, Crohn’s disease, and rheumatoid arthritis. Finally, vitamin D also seems to have the ability to block α-glucosidase, alone or in synergy with vitamins B1 and B2, with effects greater than acarbose, and this would validate its possible usefulness in the diabetic population [45,46,47].

## 3. Role of Vitamin D in Atherosclerosis

Atherosclerosis is the leading cause of cardiovascular mortality. It depends on the interaction of numerous cellular factors, types (immune system with T/B cells, monocytes/macrophages, endotheliocytes, smooth muscle cells, etc.), and notoriously hypertension, diabetes, dyslipidemias, obesity, and metabolic syndrome are considered the main risk factors. Atherogenesis is a complex and widely investigated biological process that evolves in different stages, of which chronic endothelial inflammation is undoubtedly an initial trigger. Recently, the possible role of chronic vitamin D 25 (OH) D deficiency, lower than 20 ng/mL (50 nmol/L), has emerged among the new risk factors investigated. In addition to its known role in bone metabolism (calcium/phosphorus), its possible role in cardiovascular health has been highlighted [48]. Vitamin D deficiency is present in almost 50% of the world’s population, and this figure has led many authors [8,49] to hypothesize a possible correlation with the increase in cardiovascular disease. The different mechanisms underlying this inverse relationship are based on the fact that vitamin D is directly involved in the atherosclerotic inflammatory processes and the VDRs are present in all the cells involved in atherogenesis (endotheliocytes, smooth muscle cells, immune cells, etc.). The hormonal role of vitamin D in controlling cell growth-migration-differentiation, in modulating the immune response, in inflammation and fibrosis, etc., could play a crucial part from the initial endothelial phlogistic activation phase to the vulnerability of atheroma. The hormonal action of the biologically active form of the steroidal vitamin D (1,25 (OH) 2D or calcitriol) or its analogues (hepatic metabolite or calcifediol (25OHD), etc.), biologically less active and with a shorter half-life, are linked to the action on the VDRs, known receptors in the nucleus of the cell capable of interacting directly with DNA by regulating its transcription. However, the exciting discovery that the VDRs are also localized in the caveoles of the cellular plasma membrane, in particular that of the endothelial cells, has also revealed a fast and direct (non-genomic) action of vitamin D [50]. Some authors [51] report, regarding human endothelial cells, that due to a lack of endothelial receptor activation, VDRs determine an endothelial proinflammatory state (via NF-κB signalling) with an increase in the leukocyte-endothelial cell interaction (upregulation of VCAM-1, ICAM -1 and IL-6, alteration of the rolling of mononucleate cells, etc.) capable of promoting atherogenesis. Other authors [52] cells demonstrate how vitamin D stimulates nitric oxide production in endothelial cells (NO). The relationship between vitamin D and calcifications of smooth muscle cells has been investigated [53], both in vitro and in vivo in animals with provoked terminal renal failure, highlighting that only calcitriol (1,25-dihydroxycholecalciferol) and not an analogue (para-calcitriol) determines, when incubated in cell cultures, the calcification of smooth muscle cells. This mechanism could be linked to the increase in RANKL/osteoproteregerine (OPG) expression. The activation of RANKL, in turn, triggers the hyper-transcription of nuclear factor kappa-light-chain-enhancer of activated B cells (NF-kB), a nuclear transcription factor capable of regulating the production of many proinflammatory cytokines. The vascular hyper-calcification observed in the animal was independent of the blood parathormone (PTH), and calcium-phosphorus values and calcitriol and the analogue para-caltriol determined a reduction in PTH. This study, therefore, indicated that vascular calcification was somewhat independent of the blood values of calcium/phosphorus and PTH [54], but connected to the RANKL activation pathway [55]. These notions are known in clinical practice where, in patients with chronic renal insufficiency, calcitriol (the biologically active form of vitamin D) is used to reduce secondary hyperparathyroidism, but this can lead to a greater risk of vascular calcification, and the use of para-calcitriol has shown better survival results [56]. Therefore, vascular calcifications do not seem to depend on the values of the calcium/phosphataemia [57], but are influenced by the Receptor activator of nuclear factor-kappa-Β ligand (RANKL) activation pathway belonging to the tumor necrosis factor (TNF) super-family.

Vitamin D ultimately intervenes on inflammation with various mechanisms such as inhibition of the prostaglandin/cyclooxygenase pathway, up-regulation of anti-inflammatory cytokines, reduction in adhesion molecules, reduction in the activation of metalloproteinase-9 (MMP-9 or gelatinase), and the downregulation of the renin-angiotensin system (RAS) [58,59]. The link between vitamin D and endothelial damage is identified above all in the proinflammatory nuclear factor (NF-κB) present in all cell types [58]. Epidemiological studies have shown that vitamin D deficiency can be considered a marker of cardiovascular risk [60] related to atherogenesis [61] and the consequences of acute cardiovascular events [58]. Chronic vitamin D deficiency can also trigger secondary hyperparathyroidism, increased insulin resistance (impaired pancreatic β cell function) with increased risk of metabolic syndrome, and type II diabetes mellitus (calcitriol regulates the genes involved in insulin production in the pancreas) [62]. Furthermore, vitamin D decreases the formation of foamy cells inhibiting the absorption of cholesterol from macrophages [63]. Chronic deficiency of serum 25-hydroxyvitamin D vitamin (calcidiol) has been associated with metabolic syndrome and low HDL cholesterol [64]. Vascular and endothelial smooth muscle cells, having both VDRs and the enzyme 1-α-hydroxylase, can independently produce calcitriol from calcidiol [61]. In addition, vitamin D is involved in regulating the growth/proliferation of smooth muscle cells and cardiomyocytes [61]. Vitamin D inhibits the proliferation of vascular smooth muscle cells by the acute influx of calcium into the cell. Still, renal failure increases the risk of calcification of smooth muscle cells [65]. Among the cardiovascular protective effects of vitamin D, we must include those already mentioned: anti-inflammatory effects, the inhibition of the proliferation of smooth muscle cells, the suppression of pro-atherogenic T lymphocytes, the preservation of the normal endothelial function [66], and the protection against glycation products [67]. Chronic vitamin D deficiency is associated with increased parietal vascular stiffness and, more generally, with sub-clinical atherosclerosis. Clinical trials addressing the role of vitamin D in atherosclerosis are still limited. Overall, the conflicting results yielded from experimental studies show no beneficial effects from vitamin D repletion or supplementation [68,69].

**Figure 1:**
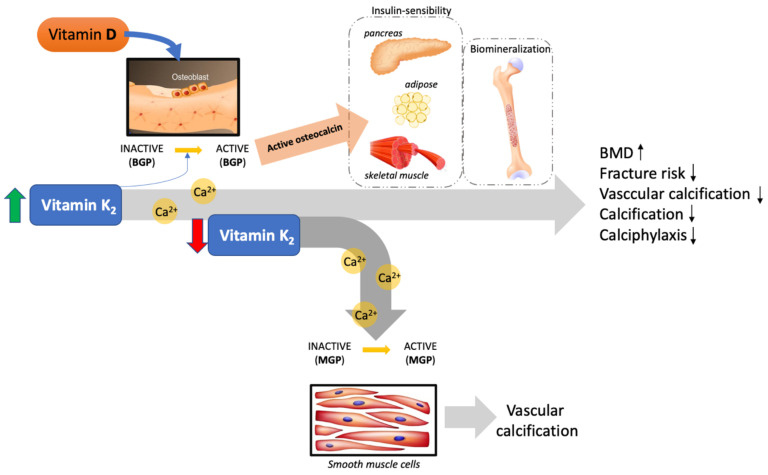
Representative image reporting the synergism between vitamin D and vitamin K. The imbalance between vitamin D and K in the blood results in a dysfunction whereby the excess calcium takes a route towards the soft tissues rather than the one leading to the bones, promoting vascular calcification. (BMP = bone Gla protein; MGP = matrix Gla protein; BMD = bone mineral density).

A new frontier of vascular research is addressing the effect of vitamin K and, in our regard, the synergic effect of vitamin D with vitamin K in the inhibition of vascular calcification (Figure 1). Recent clinical trials have not shown a beneficial effect of vitamin K in association with vitamin D concerning calcification in patients with CVD and type II diabetes [70]. These trials have various limitations, such as patient types included in the study, such as those with chronic kidney disease, drugs used by the patients at the time of the trial, and the short intervention period [71].

## 4. Role of Vitamin D in Essential Hypertension

Low plasma levels of vitamin D have been associated with an increased prevalence of hypertension [72,73] and, in particular, diastolic hypertension [73,74]. Clinical studies have shown an inverse relationship between calcitriol levels (1,25 (OH) 2D3) and values of blood pressure/renin activity in people with normal blood pressure levels [75]. The association between the polymorphisms of the vitamin D receptors (VDRs) and the risk of developing high blood pressure has also been reported [76]. The demonstration that sunlight can reduce blood pressure values by increasing circulating vitamin D, thus explaining the geographical-racial diversity of hypertensive disease, has also been highlighted [77]. Vitamin D supplementation reduces blood pressure [78] by decreasing the activity of renin-angiotensin II in patients with hyperparathyroidism [79]. Orthostatic hypotension, especially in elderly patients (risk of falls), has also been related to the chronic deficiency of vitamin D [80,81]. Vitamin D receptors are found both in vascular and endothelial smooth muscle and in cardiomyocytes, and this could be implicated in the involvement of cardiovascular adaptation mechanisms to orthostatism [82]. In some human studies, vitamin D concentration was associated with blood pressure levels in normotensive and hypertensive subjects [83]. Clinical trials with vitamin D supplementation showed a reduction in blood pressure in hypertensive and elderly patients [84,85]. A significant and inverse correlation between 25 (OH) vitamin D and blood pressure [86] was also observed in 12,000 subjects in the National Health and Nutrition Examination Survey III (NHANES III)). Additionally, Burgaz’s meta-analysis, including 18 studies, confirmed the existence of this association [87]. On the contrary, other studies do not endorse the association between vitamin D deficiency and increased susceptibility to high blood pressure [88,89]. A cause–effect relationship was established between vitamin D deficiency and high blood pressure. The Mendelian study (D-CarDia-study) extended to over 140,000 individuals of European origin divided into groups based on the type of gene coding for the protein (DBP: vitamin D-binding protein) that regulates the blood concentrations of vitamin D. The study revealed that for every 10% increase in vitamin D blood levels, blood pressure decreases and the risk of becoming hypertensive drops by 8.1%. We have previously mentioned the importance of DBP in regulating the free, biologically active portion of vitamin D and that DBP polymorphisms are very frequent, such that more than 120 have been described to date (gene coding for GC/DBP) [90]. Still, other studies, such as the ARIC study, underline the racial differences between white and black people by reimagining the role of DBP polymorphisms but reconfirming the relationship between vitamin D and coronary artery disease or even a 30% increase in the risk of PAD regardless of their ethnicity [91]. A recent meta-analysis [92] further underlines the relationship between VDR polymorphisms and increased susceptibility to hypertension [93,94]. The VDRs are encoded by a large gene (>100 kb) located on chromosome 12q12–14 and has two promoter regions and eight coding exons (2–9) and six untranslated exons (1a-1f). As mentioned in the analysis of the sequence of this gene, numerous biallelic polymorphic sites have been identified, generally for point mutations, capable of determining different cellular effects for greater or lesser transcription, an altered post-transcriptional/transductive activity, or even final modifications of the gene product (tertiary structure) [95]. The first studies on the polymorphisms of VDRs were aimed at their relationship with osteoporosis, and today the most studied are CDX2, FoKl, Apal, Taql, and Bsml, and among them, those most connected to cardiovascular pathologies and hypertension are FoKl and Bsml [92]. Ultimately, although the various studies do not agree on the role of DBP polymorphisms or VDRs, the link between chronic vitamin D deficiency and peripheral vascular pathology must be further investigated. An updated meta-analysis on clinical trials highlighted that vitamin D supplementation had no significant effects on hypertension in the general population [83]. Patients with low levels of vitamin D were not addressed, and future studies must fill this gap.

## 5. Role of Vitamin D in Peripheral Arteriopathies and Aneurysmal Pathology

Generally, the obstructive or dilating arterial diseases of the large-medium arteries are classified as peripheral arterial diseases (PAD), without considering the cerebral and coronary circulatory pathology. The problem of peripheral arterial disease is relevant for world health, as over 200 million people are estimated to be suffering from this disease worldwide [96], with the doubling of this prevalence expected by 2040 [97]. Some recent evidence shows that this disease represents a global risk in low- and high-income countries, and it is often underestimated and poorly managed [98,99]. Patients with PAD have an approximately three times greater risk of cardiovascular mortality despite the current rich availability of drug therapies [100], and in this context, all that can be done to improve the understanding of this pathology is certainly useful. The pathophysiology is multifactorial due to the frequent coexistence of several factors that synergistically contribute to the development and/or progression of PAD [101]. In addition to the known risk factors (smoking, cholesterol levels, diabetes, hypertension, metabolic syndrome, etc.), new genomic and epigenomic factors [102] have been highlighted. In particular, ten genes have proven to be particularly relevant as possible new biomarkers (NNMT, LUM, ITLN1, CFD, TSPAN8, SERPINA5, MMP28, GPC3, GDF10, and FDXR) [103,104]. The discovery of the presence of receptors for vitamin D in the vascular system has led researchers to investigate a possible role in the pathophysiology of peripheral arterial diseases [105]. The issue of vitamin D deficiency in the general population (50%) is well known, and further studies such as Higler’s [106], in which 195 studies published over the period 1990–2011 were reviewed, with over 168,000 cases highlighted the distinct geographical differences regarding vitamin D blood values among the peoples of North America (higher values) compared to Europe, the Middle East, and Africa, or even the differences between the individual European nations [107], and this was related both to race and the availability of sunlight. Another noteworthy element is the reduction with age in the synthesis capacity of vitamin D in the skin, due to sunlight and the progressive decrease in VDRs’ muscle receptors in the elderly with the possible implication of sarcopenia [108]. As a result, older adults suffer from lesser vitamin D availability compared to young individuals, as the skeletal muscle gradually loses VDRs with age. Many studies [88,109,110,111,112,113,114,115,116,117,118] show a strong correlation between the reduced levels of circulating vitamin D and the presence of peripheral arterial disease, although other studies have not validated this possibility. It should be noted that the cut-off for hypovitaminosis D was very different in the different studies (range <17.8 ng/mL to <30 ng/mL), and this can represent a factor of confusion and contradiction between the various findings as well as the different geographies of the populations involved. Data from the first National Health and Nutrition Examination Survey (NHANES with 4.864 participants over the period 1999–2002) showed that patients with PAD had lower plasma levels of vitamin D. Additionally, the second National Health and Nutrition Examination Survey (2001–2004) showed a significant prevalence (>80%) of PAD in patients that had the lowest values of vitamin D [119]. The recent meta-analysis of six observational studies also showed that patients with PAD had lower vitamin D values than those without PAD (controls group) [120]. Vitamin D levels appeared to be reduced in the more advanced stages of vasculopathy. Other studies have also highlighted the relationship between greater Ankle Brachial Index (ABI) reduction and lower circulating vitamin D levels [121,122]. Ultimately, the chronic decrease in vitamin D levels in patients with PAD can depend on several factors, such as the lower capacity of solar photosynthesis with skin ageing, the reduction with age in VDRs, nutritional deficits, race, reduced sun exposure, etc.

The various biological actions of vitamin D on the vessel wall are attributable to its effects on one hand on the calcium-phosphorus metabolism, and on the other on the renin-angiotensin system (RAS), which is known to regulate blood pressure, circulating plasma volume and the tone of the arterial muscle [123,124], accompanied by the ability to increase NO [125] production, which in turn can regulate the renin-angiotensin system (RAS) [126] and the immune system.

The role of vitamin D has also been studied in aneurysmal dilated arterial disease. The progressive degeneration of the arterial wall secondary to a chronic inflammatory process can determine the slow decline and remodeling of the extracellular matrix, the release of elastin peptides, etc. All of these are pathogenic mechanisms of progressive arterial dilation [101,127]. Abdominal aortic aneurysms affect 4–8% of men, and approximately 1.5% of cases in both males and females after 60 years of age remain asymptomatic until rupture, which is an emergency with a high mortality rate (80–90%). We know that, for example, abdominal aortic aneurysms affect about 20 million people worldwide and that with the increase in the average age, there will be a further increase in their prevalence in the near future. Additionally, recent proteomics studies identified possible new predictive biomarkers such as plasma bleomycin hydrolase (BH) and others to help identify subjects with an abdominal aneurysm at greater risk of rupture [128]. In the literature, there are numerous reports of chronic hypovitaminosis D being associated with a high incidence of abdominal aortic aneurysms, although there are other studies that do not support this hypothesis [129,130].

An example is the randomized observational study by Wong et al. [131] on a large group of 4233 older adults (age 70–88 aa). It pointed out that low vitamin D values are indeed associated with larger aneurysms. Another study, but on a small sample (11 patients), demonstrated how the selective activation of the VDRs with the paricalcitol interferes with inflammation-mediated by calcineurin. Another proteomic-redox study highlighted the possible role of carbonylation of specific serum proteins (serum retinol-binding protein (RBP), vitamin D-binding protein (DBP), fibrinogen α-chain HNE (protein-bound 4- hydroxy-2-nonenal)) particularly present in patients with abdominal aortic aneurysm compared to controls, implying that these proteins are essential in the management of oxidative stress. Other interesting aspects are based on the in vitro observation that the activation of the endothelial VDRs with calcitriol (1,25-dihydroxycholecalciferol) inhibits both the activation of angiotensin II and the production of proinflammatory and angiogenetic endothelial chemokines caused by leukocyte–endotheliocyte interaction and, as verification, the abolition of VDRs negates this effect of calcitriol [132]. There is still debate as to whether free vitamin D levels (not related to carrier proteins) are more important than those currently considered to be more reliable biomarkers than the actual state of vitamin D [133].

Future studies may clarify these important aspects. Some studies have highlighted aspects concerning the role of vitamin D in the “atherosclerotic plaque” [134], in which the interesting correlation between low circulating levels of vitamin D and atheroma was underlined. These studies investigated whether the evolution of atheroma could reduce the expression of the VDRs, an aspect not yet fully clarified. Dendritic cells (DC) are a class of immune system cells capable of capturing and exposing the antigen. In partnership with the monocytes/macrophage system, they have had considerable attention in recent years due to their role in atherogenesis. The interaction via the VDRs of these vascular wall dendritic cells would have an important regulatory role in the atherogenic process [135,136]. Other aspects still being discussed are the relationship between vitamin D deficiency and statin therapy induced musculoskeletal disorders, and type of statin relative to circulating levels of vitamin D [137,138]. These observations are based on the fact that statins can reduce cholesterol levels and 7-dehydrocholesterol (provitamin D3), the cutaneous precursor of vitamin D.

Ultimately, the role of vitamin D in the immune system seems to be fundamental in atherogenesis and the development of related diseases (PAD, etc.). It has modulating effects on innate and adaptive immunity (it favors innate and inhibits adaptive immunity), namely the ability to promote the expression of antiatherogenic monocytes-macrophages [139], and its anti-inflammatory-immunomodulating activity in systemic inflammation [58]. In particular, the immunosuppressive activity of vitamin D is mediated by the reduction in inflammatory cytokines (interleukin (IL-2,6), interferon ϒ (IFN-ϒ), tumour necrosis factor (TNF), etc.). The immunomodulating activity is mediated both by the enhancement of regulatory T cells (important in autoimmunity) and in the induction of the transcription of FOXP3 (forkhead box P3 or scurfin), important in the control of regulatory T cells [140]. The Atherosclerosis Prevention in Pediatric Lupus Erythematosus (APPLE) study showed, with a 3-year follow-up, a slower progression of carotid atherosclerosis (CIMT: carotid intimate medial thickness) in patients treated with atorvastatin and with vitamin D levels ≥ 20 ng/mL compared to those who had vitamin D values ≤ 20 ng/mL (295) and also a greater reduction in PCR-ultra-sensitivity (hsPCR: high-sensitivity C-reactive protein) [141].

## 6. Genetic and Epigenetic Role of Vitamin D

As already mentioned, the key points in the metabolism of vitamin D are hepatic 25-hydroxylation (mitochondrial and microsomal) by the cytochrome P450 enzyme (family 2-subfamily R member 1-CYP27A1) and subsequently, at the renal level, 1–25-hydroxylation by the cytochrome P450 enzyme (cytochrome p450 27B1-CYP27B1) with the formation of biologically active vitamin D (calcitriol), which acts at the level of VDRs (nuclear-cytosolic, located in humans on chromosome 12q12-q14), similarly to a steroid receptor. Genetic studies have clarified the genomic action of vitamin D for its transcriptional regulation capacity, which occurs through a fundamental, intermediate step of formation of a heterodimer with retinoid X receptor (RXR). Subsequently, the genomic action is carried out on the DNA sequence called vitamin D response element (VDRE) in the region of promoter genes regulated by vitamin D [142]. We have already mentioned the different VDR polymorphisms that can account for the different circulating rates of vitamin D. Today we know that about 3% of the human genome is influenced by vitamin D [143]. Epigenetics can be defined as the set of inheritable changes in gene expression not caused by changes in the DNA sequence, therefore changes that end up influencing the phenotype without altering the genotype. We can imagine epigenetics as a sort of interface between our genome and the world outside the cell capable of perceiving any changes, which, if repeated or chronic, can eventually change the gene expression and pass it on to the descendants without altering the nucleotide sequence of a gene, only its activity [143]. Therefore, an epigenetic signal is a possible external factor such as lifestyle, stress, nutrient deficiency, obesity [144]. Among the different epigenetic mechanisms capable of modifying gene expression, the modification of histones (basic proteins constituting the chromatin and organized to form the nucleosome in groups of 8 units) and histone methylation are the most important mechanisms. Histone acetylation/deacetylation (HAT: histone acetyltransferase; HDAC: histone deacetylase) in dynamic equilibrium determine the activation or deactivation of transcription (histone acetylation generally correlates with transcriptional activation, effect “ON “). The primary epigenetic effects of vitamin D are linked precisely to the ability to promote chromatin reading and transcription through histone modifications (acetylation). It has been shown that the VDRs-RXR dimer can interact directly with HAT by inducing transcriptional activation [145,146]. Most chromatin sites of action of vitamin D are present in the region of chromosome 6, which hosts the human leukocyte antigen (HLA) or major histocompatibility complex (MHC) cluster, which is essential in the immune response, and this underlines the epigenetic immune activity of vitamin D [147]. Some in vitro studies [148] show that 2 months of 10 µg/50 µg daily supplementation of vitamin D3 (Cholecalciferol) determines a significant regulation of 291 genes. In contrast, higher doses (500 µg/day) in young subjects under 35 determine significant changes in 99 circulating mononuclear cell genes (PBMC: Peripheral Blood Mononuclear Cell) compared to placebo. Even boluses of vitamin D (2000 μg every 28 days, 3 times—vitamin D-bolus intervention trial) in a healthy subject showed that slight increases in serum vitamin D levels could significantly modify hundreds of sites within the human leukocyte epigenome [149]. The epigenomic mechanism of methylation is mainly meant to inhibit gene expression and genomic integrity with surveillance of any “parasite sequences” (viruses, etc.). Cytosine methylation maintains chromatin in a condensed form, blocking the transcription process. Severe vitamin D deficiency appears to be associated with methylation changes in leukocyte DNA [150] and circulating levels of vitamin D related to the degree of DNA methylation.

## 7. The Role of Vitamin D in Cardiac Remodelling and Disease

The link between vitamin D and cardiovascular function, as partially described previously, involves the renin-angiotensin system (RAS). Increased renin kidney levels and higher plasma angiotensin II and aldosterone levels are detectable in VDR -/- mice [151]. Administration of 1,25-OH D can reverse this, acting as a negative regulator of the renin gene transcription [152]. In the literature, excessive RAS activation has been extensively shown to cause hypertension and cardiac hypertrophy [153]. Mice with VDR -/- and with lack of 1-alpha-hydroxylase show both of these alterations, and as before, they can be readily reversed with vitamin D supplementation [154].

Moreover, vitamin D deficiency causes not only cardiomyocyte hypertrophy but also increased myocardial collagen deposition [155]. This is due to RAS activation on the one hand and VDR impairment on the other [156,157]. Mice with VDR deletion develop myocardial hypertrophy regardless of hypertension, excess RAS activation, or hypocalcemia [158]. This independent effect of vitamin D is further supported by the fact that hyperparathyroidism and hypercalcemia correction does not improve such cardiac abnormalities [154]. Another important finding is the association between vitamin D and Ca2+ management in cardiomyocytes, which seems to have a beneficial effect, leading to increased systolic and diastolic heart function [159]. Vitamin D deficiency is also correlated with cardiac steatosis, and VDR gene knockout mice display increased interstitial fibrosis with overexpression of collagen (1α1, 3α1) and matrix metalloproteinase (MMP-2), leading to cardiomyopathy and HF [160]. These alterations are attenuated with vitamin D supplementation [161].

These experimental models find confirmation in population-based studies [162]. Vitamin D deficiency is strongly correlated with hypertension and left ventricle hypertrophy (LVH) [163]. In particular, insufficient exposure to the sun and consequent vitamin D deficiency has been associated with LVH and Fabry’s cardiomyopathy [164]. Although dilated cardiomyopathy (DMC) is mainly idiopathic, studies have investigated its correlation with vitamin D due to maternal deficiency. In this case, maternal vitamin D deficiency caused hypocalcemia, and consequent hypoparathyroidism was the cause of impaired cardiac function, DCM, and ultimately HF [165,166]. These infants with rickets and cardiac impairment greatly benefited from vitamin D and calcium supplementation, further confirming the importance and the role of this vitamin [167,168]. Vitamin D has also been used as a predictor for CV mortality in heart failure [169].

Additionally, vitamin D receptor (VDR) polymorphism and expression have also been used as potential risk factors for cardiovascular diseases such as cardiomyopathy and HF [170]. Overall, vitamin D is associated with cardiovascular events, as shown by an analysis of patients with MI which almost all had low vitamin D plasma levels [171]. More importantly, vitamin D deficiency is associated with poor patient outcomes with acute coronary syndrome (ACS) [172]. This is partially due to the inverse relationship between hyperlipidemia and vitamin D. Low levels of vitamin D coincide with hyperlipidemia and hyper-homocysteine [173]. Atherosclerosis and pro-inflammatory state play a fundamental role in cardiac disease genesis as addressed previously [174,175]. Taken together, all of these diseases lead the way to heart failure (HF), and thus vitamin D deficiency is associated directly and indirectly with this path [176]. The therapeutic role of vitamin D in heart diseases and HF was highlighted in the VINDICATE study, and those studies with controversial results may be due to genetic VDR variation and implication [177,178]. Lastly, vitamin D has also been associated with rhythm abnormalities and atrial fibrillation (AF) [179]. Vitamin D deficiency and extensive left atrial fibrosis increase AF incidence and AF recurrence [180]. This association, however, is not independent and is always accompanied by either HF or coronary artery bypass, and further investigations are necessary [181,182]. As mentioned before, current clinical trials and meta-analyses do not confer cardiovascular protective properties to vitamin D [183]. No significant correlation has been highlighted between vitamin D and left ventricle functions and/or atrial fibrillation in the general population or post-operative settings [184,185,186].

## 8. Conclusions

Studies view vitamin D increasingly as a hormone, confirming its implication in multiple chronic pathologies (atherosclerosis, diabetes, metabolic syndrome, heart disease, immune, etc.). Not only that, but its well-known regulatory function on bone metabolism is also today a burgeoning sector of research worldwide. As of today, observational studies, meta-analyses, and clinical trials are scarce and full of limitations. Doubt remains as whether vitamin D deficiency can be used as a marker of pathology or is a pathology itself. Findings from the molecular level up to the clinical level do not overlap and frequently disagree. Trials are contradictory as to which type of vitamin D is best suited for supplementation.

There are conflicting results between observational studies regarding VDR activators in patients with end-stage renal disease and increased vascular calcification [187]. A comparative study showed that calcitriol and paricalcitol in doses for correcting secondary hyperparathyroidism had protective effects against aortic calcification, while higher doses stimulated aortic calcification [187]. This to say that the correct dosage of calcitriol could protect against disease-induced vascular calcification, but further efforts are needed [187].

The role of vitamin D in pathologies that represent cardiovascular risk factors did not support vitamin D as a prevention strategy [30,31]. Diabetes mellitus, metabolic syndrome, and obesity correlate with vitamin D deficiency, but no beneficial effects from supplementation have been observed [42,43]. In fact, despite a large amount of positive preclinical evidence, clinical trials and comparative meta-analyses also rejected vitamin D supplementation in atherosclerosis, cardiac remodeling, hypertension, and peripheral arteriopathies [83,84,100].

Given the current state of the art, much is to be done. Larger, longer, and better-designed studies are needed to understand the gap between preclinical and clinical studies. Addressing the implications related to vitamin D is important for the well-being of a large population.

## Figures and Tables

**Table 1:** Table reporting the presence of 1-α-hydroxylase and vitamin D receptor (VDR) in different apparatus/system.

Apparatus/System	1-α-Hydroxylase	Vit D Receptor (VDR)
**Endocrine**		
pancreatic β cells (insulin)	+	+
Parathyroid Cells	+	+
Thyroid/adrenal/pituitary cells	−	+
**Cardiovascular**		
Myocardiocytes/Endothelium	+	+
Smooth muscle cells	−	+
**Skeletal Muscle**		
Cartilage, chondrocytes, osteoblast	+	+
Skeletal muscle fibres	−	+
**Gastro-Intestinal**		
GALT (Gut Associated Lymphoid Tissue)	+	+
Esophagus-stomach-intestine	−	+
Liver	−	+
**Genitourinary**		
Prostate	+	+
Testis, ovary, uterus	−	+
Breast-placental/Decidual	+	+
**Nervous**		
Neurons-Glia	+	+
**Blood and Immune**		
Macrophages, monocytes, lymphocytes (B-T) dendritic cells	+	+
Bone marrow	+	+
Thymus	−	+
**Integumentary**		
Keratinocytes	+	+
Hair follicle cells	−	+
**Adipocytes**	−	+
